# Effects of Household Size and Partner Status on Financial Well-Being and Financial Exploitation

**DOI:** 10.1093/geroni/igaa057.377

**Published:** 2020-12-16

**Authors:** Jessica King McLaughlin, Elizabeth Anderson, Ashley Taeckens-Seabaugh, Jennifer Greenfield, Eric Chess, Jessica King

**Affiliations:** University of Denver, Denver, Colorado, United States

## Abstract

Social isolation and loneliness are significant risk factors for older adults (adults over the age of 50) for a multitude of reasons. This study explored the connections between social isolation, vulnerability to financial exploitation, and financial wellbeing in a sample of older adults. Using the concept of precarity as a guiding framework, this study assessed behaviors that expose individuals to financial exploitation as well as indicators of financial wellbeing in individuals with varying degrees of social isolation. One-hundred-eight community-dwelling older adults completed a pen-and-paper survey containing measures of risk to financial exploitation and financial wellbeing. Participants were predominantly female (75.9%), White (90.7%), and highly educated. Fifty-eight participants were unpartnered (53.7%) and 67 participants (62%) lived with at least one other person. Chi-square analyses resulted in a significant association between partner status and being a victim of financial exploitation (χ2(1) = 10.842, p≤.001). Furthermore, there was an association between social isolation and engaging in certain behaviors that put someone at risk for financial exploitation (such as reading junk mail). As for measures of financial wellbeing, partner status was significantly associated with feelings of financial freedom (χ2(3) =8.608, p =.035). These findings indicate that socially isolated individuals may be at greater risk of financial exploitation and have lower levels of financial wellbeing, such as feeling unprepared for retirement (χ2(2) =7.358, p =.025). The results of this study highlight the risks involved with social isolation and indicate further research is needed into ways to mitigate these negative effects.

